# December

**Published:** 1890-12-06

**Authors:** 


### DECEMBER.

Bright crisp frost raises the spirits and sets the blood in healthy circulation by provoking exercise. And bright crisp frost we have a right to expect in December, although it is unfortunately true that we do not always get it. On the contrary, December is often characterised by damp, cold, and drizzle, sometimes by snows and thaws, and especially towards the end of the month by easterly winds. Martial applies to December the epithet *canus* or hoary. "Great things now doeth the surpassing knowledge. Behold! He saith to the snow be, and on the earth it falleth!" Our Saxon ancestors called December the winter-moat (month), or yule month, from "zeppeol," a wheel, emblematic of the sun's revolution and return to his course—the 22nd of December being the date of the winter solstice, when the sun reaches the tropic of Capricorn. But after becoming Christians the Saxons called December "heligh-moat" or holy month, because Christmas fell within it. The name December is not etymologically correct for in the old Roman calender the year was divided into ten months, the last being called December or tenth. But the name was retained when the year was divided into twelve months. The Roman Emperor Commodus attempted to change the names of several of the months, and gave "Amazonius" to December, in honour of his mistress Martia, who clothed in Amazonian attire. But Commodus was profligate, and eventually he was poisoned, so the names of the months were not altered. The Saturnalia or great festival of the god Saturn, whom the Romans identified with the Greek Cronus, took place in December. It was a time of general mirth and joy. Distinctions of rank were temporarily laid aside, gambling, at other times illegal, was permitted, and tradition has it that human sacrifices were offered. There is no doubt that the origin of the Saturnalia was a celebration of the winter solstice. But like various other festivals, of which we have examples in India, it came to mean something else. There are few, if any, instances of festivals retaining their original purity and meaning in any other religion than the Christian. And of all cults, probably Hinduism sins most in this respect, in proof of which we have only to mention the Hindu Holee. In this connection we note having seen in the streets of London on the 5th of November last, several figures of "Jack the Ripper" instead of Guy Fawkes. December 6th is known as St. Nicholas' day. Nicholas was a saint of great virtue. It is stated that an Asiatic gentleman sent two sons to the saint for education. The innkeeper where the boys lodged, cut them to pieces, pickled them, and intended to sell them for pork. This was revealed to Saint Nicholas in a dream, who thereupon taxed the innkeeper with the crime, put the pieces together, and restored the youths to life. An old writer observes, "If there were no other miracle, the pickled children would be sufficient to establish Nicholas' fame as the patron of youth." On this date it was the custom of the choir boys of cathedrals to choose their "boy bishops," whose authority lasted till the 28th. Stone monuments to these "boy bishops" have been discovered at Salisbury and other places. Borlase, in his "Antiquities of Cornwall," mentions as a relic of Druid fancies, sleeping on stones on the night of December 6th as a cure for lameness! December 8th, 1824, is notable as the date of an earthquake at Chichester, when in the newspapers of the period the usual accounts appeared. On December 10th, 1658, the first stage coach was established from Aylesbury to London. Shortly afterwards a writer said: "By stage coaches one may be transported to any place, sheltered from foul weather and foul ways, free from endangering one's health or body by hard jogging or over-violent motion; and this not only at a low price, as about a shilling for every five miles, but with such velocity and speed, as that the posts make not more miles in a day; for the stage coaches called flying coaches make forty or fifty miles in a day." But many people in those times, and of "the better rank" also, still journeyed by waggon at the rate of twelve miles *per diem!* On December 12th, 1653, Oliver Cromwell was declared Protector, and on the same date in 1688 James II. abdicated. On December 12th, 1621, a writer said: "Of all trades in the world a brewer's is the loadstone which draws the customs of all functions into it. It is the mark or upshot of every man's ayme, and the bottomlesse whirlepool that swallows up the rich and poor." We have heard a good deal said against brewers and publicans of late, but the majority of English people will have their beer—the modern representative of the old Saxon mead—so we presume brewers and publicans will still exist, notwithstanding any outcry against the class, or any diminution of their privileges. On November 14th, said another old author: "Happiness now spreads throughout the country like an epidemic, and all parties agree in preparing to greet old Christmas with cordial welcome." But this was in the "good old times," and we doubt if an epidemic of happiness now spreads over the land on the approach of Christmas. To the elderly it is rather a sad period, for "a voice comes o'er the waves of time," recalling to memory the many who have departed before us. December

17th was a reputed unlucky day especially to give a knife away, "for knives they tell me always sever love." By this time all good old housewives were supposed to have laid in their stores for the Christmas festivities, also to have made arrangements for the "roast beef of England" by training the "turnspit" dog. This was effected by putting the dog in a wheel, and a burning coal behind him! The dog could not stop without burning his legs, so was obliged to move on! On December 22nd, 1753, died Mr. Braitwaite, of Carlisle, aged 110, who for one hundred years sang in the Carlisle Cathedral. This brings Christmas carols to mind. The word is said to be derived from *cantare*, to sing, and *rola*, an interjection of joy. It is creditably reported that the ancient bishops caroled amongst their clergymen on the approach of Christmas. Now, Christmas carols are chiefly used as a means of extracting alms from the charitable! Of December 24th (Christmas Eve) we find in Batesman's Treasury (1707) the following lines:—

"Up Dolly, Peg, Susan, you spoke to me,
Betimes to call you, and its now past three.
Get up on your buts ends, and rub your eyes,
For shame! No longer lye a bed, but rise,
The pewters to scower, the house to clean,
And you abed! Good girls! What is it you mean."

We wonder what the modern Jane, or Sarah, or Mary-Anne, would say if roused at 3 a.m., even on Christmas Eve? Of December 25th, Christmas Day, quires might be written. We must, however, be brief. We must pay no attention to "memory, celestial maid, who glean'st the fragments crossed by time." Now, evergreens and winter flowers are real friends, not forgetting the mistletoe, "sweet emblem of returning spring"; under which "many a maiden's cheek has reddened been!" "With footsteps slow, in furry pale, his brows enwreathed with holly, old Christmas comes to close the waning year. Upon a shaggy bearded goat he rides, and in his hand a broad deep bowl he bears, of which he freely drinks." Of Christmas festivities we will only refer to the "boar's head," for "ye bore ys a souveragn beste, and acceptable in enny feste," and now, "no more cruel than the orange in its mouth." We remember an old Oxford Christmas song illustrating the combat of the Oxford Don with the "mighty boar."

"So dreadful this bristle-back'd foe did appear,
You'd have sworn he had got the wrong pig by the ear.
But instead of avoiding the mouth of the beast,
He rammed in a volume and cried, *Græcum est!*"

December 26th is Boxing Day, when of yore as now, the employés of all grades "from their master's customers implore the yearly mite." And December 28th is Innocents Day, when, "behold, the Angel of the Lord appeared to Joseph in a dream, saying Arise! and take the young child and its mother and flee into Egypt, for Herod will seek the young child to destroy him."

"From Herod's cruel order they,
By Angels orders fled away,
And painters add, an Angel too,
Attended them the journey thro'."

In concluding these notes of the months, we may be permitted to wish the readers of The Hospital "a merry Christmas and a happy New Year." But a due and proper feeling of our insignificance in the scale of creation is essential. The revolution of this sphere of ours in its annual orbit round the 95 millions of miles distant sun, will soon once more introduce the month of January to us. Let us therefore recollect that since this time last year, without any effort of our own, this earth has conveyed us through space six hundred millions of miles, at the rate of 68,000 miles per hour; or one thousand times quicker than the fastest express train ever sped. Yet here we have arrived without accident. Surely this is cause for congratulation, and for serious contemplation at this season, when thoughts naturally turn to the attributes of an over-ruling Providence.

